# Hypomyopathic Dermatomyositis Presenting with Erythroderma and Concomitant Sjögren’s Syndrome: A Rare Case Report

**DOI:** 10.5152/ArchRheumatol.2025.11170

**Published:** 2025-09-01

**Authors:** Haluk Cinakli, Gülay Alp, Gülden Diniz, Dilek Solmaz, Servet Akar

**Affiliations:** 1 Department of Rheumatology, Kırklareli Education and Research Hospital, Kırklareli, Türkiye; 2 Department of Rheumatology, Uşak University, Uşak, Türkiye; 3 Department of Pathology, Izmir Demokrasi University, İzmir, Türkiye; 4 Department of Rheumatology, Izmir Katip Çelebi University, İzmir, Türkiye

Dear Editor,

Dermatomyositis (DM) is an uncommon autoimmune disorder affecting the muscles, resulting in weakness and accompanied by a skin rash.^1^ While most cases exhibit muscle and skin symptoms, other forms of the condition also exist. Clinically amyopathic DM is characterized by typical skin manifestations seen in DM without accompanying muscle weakness and is subdivided into hypomyopathic DM (HDM) and amyopathic DM (ADM). Patients diagnosed with HDM exhibit no clinical signs of muscle weakness. Nonetheless, laboratory tests, electromyography (EMG), magnetic resonance imaging (MRI), or muscle biopsies may reveal evidence of myositis. Conversely, ADM patients show no clinical, laboratory, imaging, or pathological evidence of muscle involvement.^2^

Erythroderma, also known as exfoliative dermatitis, is a severe dermatological condition that can be life-threatening. It is characterized by widespread erythema and scaling affecting over 80% of the skin’s surface area.^3^ According to a prospective study, common underlying etiologies include eczema (20.7%), psoriasis (16.8%), Sézary syndrome (12.3%), drug eruptions (12.3%), atopic dermatitis (8.7%), and mycosis fungoides (5.5%).^4^ To date, erythroderma in DM has been reported only rarely and almost exclusively in adults.^5,6^ However, no cases of HDM presenting with erythroderma have been reported. This report presents a case of HDM presenting with erythroderma accompanied by Sjögren’s syndrome (SjS).

An 88-year-old female patient was referred to the clinic by the dermatology department due to histopathological findings on a skin biopsy consistent with DM. Verbal informed consent was obtained from the patient. She presented with a diffuse red skin rash accompanied by generalized itching. Her medical history did not reveal any significant findings. On physical examination, widespread cutaneous erythema was detected on her upper extremities, lower extremities, and trunk (Figure 1). Additionally, manual muscle testing revealed normal muscle strength in all extremities. The laboratory results were as follows: hemoglobin, 11.6 g/dL; leukocyte count, 5420/mm^3^ with 6.7% eosinophils; erythrocyte sedimentation rate, 64 mm/h. Normal serum chemistry levels, including creatine kinase 111 (28-168 U/L) with only a slightly elevated lactate dehydrogenase, 329 IU/L (0-246 IU/L). The serological tests for antinuclear antibodies, antibodies to extractable nuclear antigens, antineutrophil cytoplasmic antibodies, rheumatoid factor, and anti-cyclic citrullinated peptide were negative. Additionally, tumor markers, including CEA, CA 19-9, CA 125, α-fetoprotein, and β2-microglobulin, were also negative. A contrast-enhanced whole-body computed tomography (head, neck, chest, abdomen, and pelvis) scan performed at baseline revealed a 2 cm polypoid lesion at the gastric cardia. Subsequent upper endoscopy with targeted biopsy demonstrated hyperplastic mucosal changes and chronic gastritis without evidence of intestinal metaplasia or dysplasia. No additional suspicious lesions were identified on imaging. Considering the potential association of erythroderma with hematologic malignancies, a hematology consultation was also requested. However, no hematologic malignancy, including Sézary syndrome, mycosis fungoides, or adult T-cell leukemia/lymphoma, was identified during the evaluation. A skin biopsy showed vacuolar interface dermatitis and dermal mucinosis. A minor salivary gland biopsy was performed due to the patient’s dry mouth and eyes. There was focal sialoadenitis with a focus score ≥1, and the histomorphological findings were compatible with SjS. Capillaroscopy showed disrupted capillary architecture with giant (>50 µm), bushed, and ectatic capillaries (20-50 µm) (Supplementary Figure 1). Although the patient had normal muscle strength and enzyme levels, needle EMG and MRI of the upper and lower extremity muscles were performed to exclude HDM, as the skin biopsy findings were consistent with DM. Based on these imaging and electrophysiological findings, a muscle biopsy was obtained from the deltoid muscle. In the striated muscle biopsy sent, size-shape differences and degeneration and regeneration-like injury findings were observed in the myofibers. Dense perimysial lymphocytes were noted in some areas, and a peri-fascicular atrophy-like appearance was observed. In Masson trichrome, the increase in fibrous tissue in the interstitium, indicating a chronic event, was minimal. There were pathological, immature fibers in the perifascicular groups, with neonatal myosin. Additionally, type 2 myofiber atrophy was observed with fast myosin (Supplementary Figure 2). These histopathological findings were evaluated as compatible with DM. We began systemic glucocorticoid therapy with prednisolone (1 mg/kg) and azathioprine. The erythroderma gradually resolved. During the 2-year follow-up of the patient, no loss of muscle strength or malignancy developed.

Although erythroderma has been reported in AMD, the patient’s muscle biopsy showed histopathologic features consistent with DM despite normal strength, confirming HDM. This distinction matters because HDM may warrant closer monitoring and systemic therapy, and it carries different prognostic implications. As a result, even if there is no clinical loss of muscle strength in a patient presenting with erythroderma, a diagnosis of DM should also be considered.

## Supplementary Materials

Supplementary Material

## Figures and Tables

**Figure 1.**
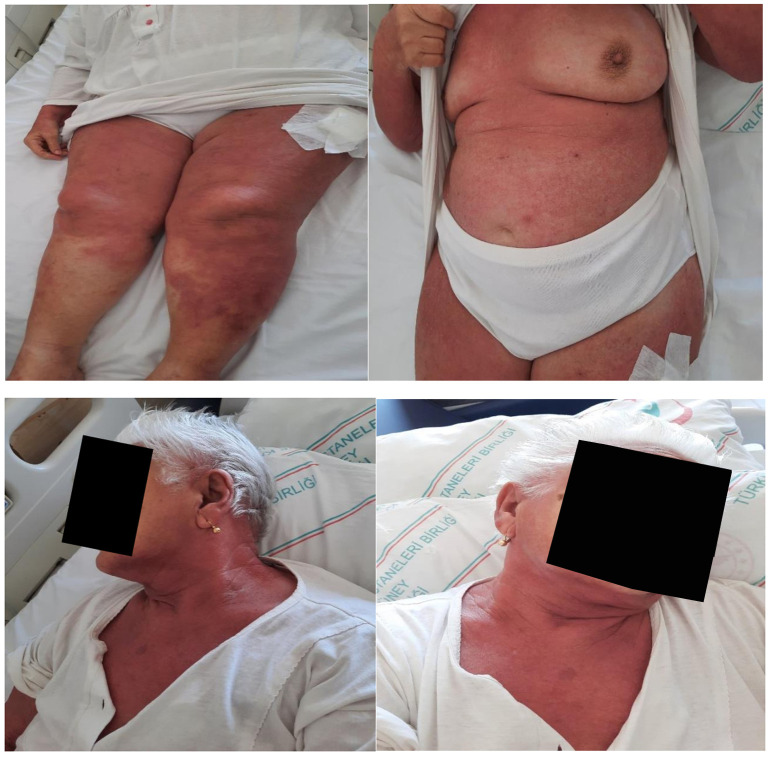
A widespread erythematous lesion on the upper extremities, lower extremities, and trunk.

## Data Availability

The data that support the findings of this study are available on request from the corresponding author.
