# Supplementary material for: Hypomyopathic Dermatomyositis Presenting with Erythroderma and Concomitant Sjögren’s Syndrome: A Rare Case Report
Source: Arch Rheumatol. 2025 Sep 1;40(3):407–9. doi: 10.5152/ArchRheumatol.2025.11170 (PMC12502843; doi:10.5152/ArchRheumatol.2025.11170)
Supplement: Supplementary Material [file supplementary_material.pdf]

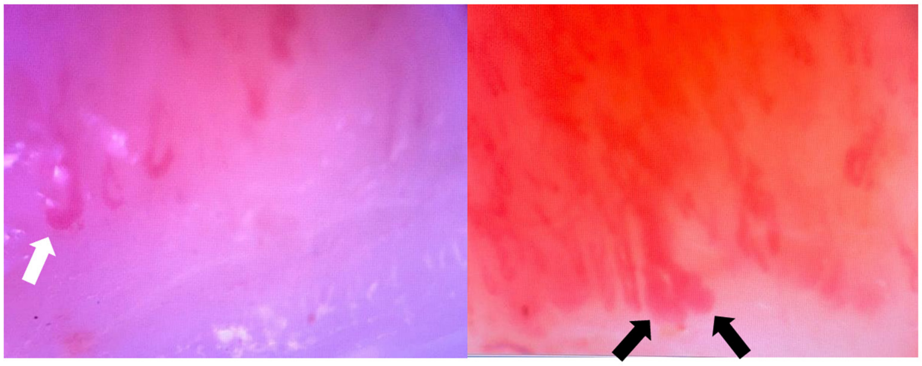

Supplementary Figure 1. Capillaroscopy showed disrupted capillary architecture with giant(>50 μm), bushed and ectatic capillaries (20 – 50 μm)> Black arrows indicate giant capillaries, and white arrows indicate ectatic capillaries.

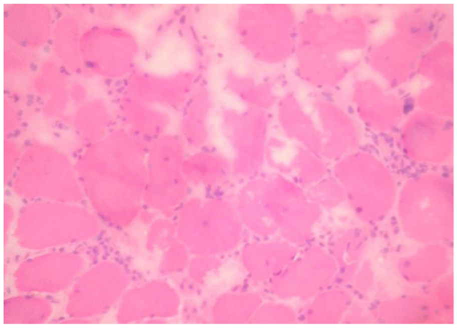

Size and shape difference in fibers.

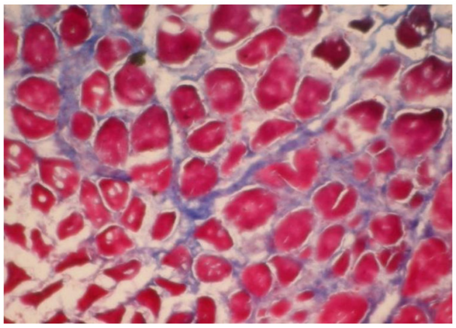

Slightly increased interstitial tissue in Masson trichrome

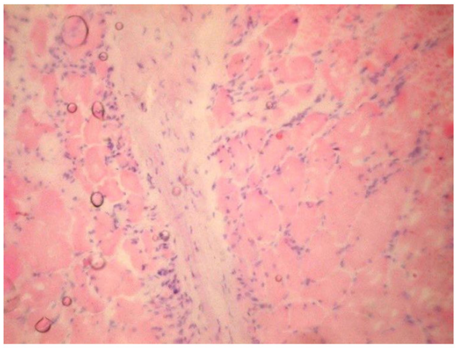

Perifascicular atrophy.

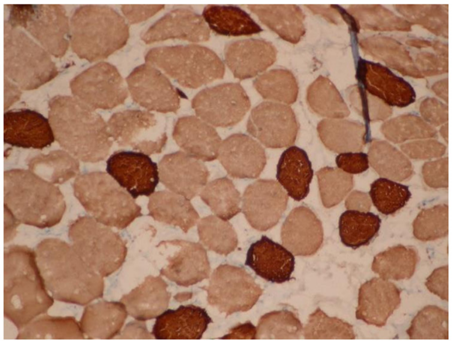

Type 2 myofiber atrophy with fast myosin.

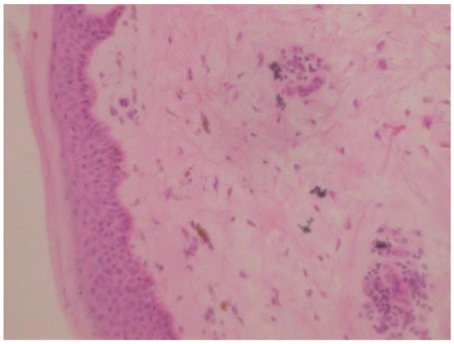

Perivascular lymphocyte infiltration under the skin.

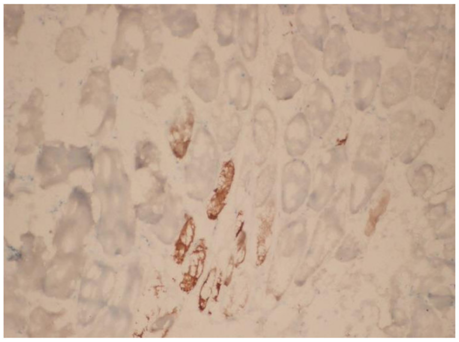

Numerous pathological immature fibers (neonatal myosin).

Supplementary Figure 2. Muscle biopsy pathology findings.
